# Extrinsic and Intrinsic Frequency Dispersion of High-*k* Materials in Capacitance-Voltage Measurements

**DOI:** 10.3390/ma5061005

**Published:** 2012-06-01

**Authors:** J. Tao, C.Z. Zhao, C. Zhao, P. Taechakumput, M. Werner, S. Taylor, P. R. Chalker

**Affiliations:** 1 Department of Microelectronics, Xi’an Jiaotong University, Xi’an 710016, China; E-Mail: tj.19861225@stu.xjtu.edu.cn; 2 Department of Electrical and Electronic Engineering, Xi’an Jiaotong-Liverpool University, Suzhou 215123, China; E-Mail: chun.zhao@liverpool.ac.uk; 3 Department of Electrical Engineering and Electronics, University of Liverpool, Liverpool L69 3GJ, UK; E-Mails: pooh@liverpool.ac.uk (P.T.); m.werner@liverpool.ac.uk (M.W.); s.taylor@liverpool.ac.uk (S.T.); 4 Department of Materials Science and Engineering, University of Liverpool, Liverpool L69 3GH, UK; E-Mail: pchalker@liverpool.ac.uk

**Keywords:** high-*k* dielectrics, frequency dispersion, dielectric relaxation

## Abstract

In capacitance-voltage (C-V) measurements, frequency dispersion in high-*k* dielectrics is often observed. The frequency dependence of the dielectric constant (*k*-value), that is the intrinsic frequency dispersion, could not be assessed before suppressing the effects of extrinsic frequency dispersion, such as the effects of the lossy interfacial layer (between the high-*k* thin film and silicon substrate) and the parasitic effects. The effect of the lossy interfacial layer on frequency dispersion was investigated and modeled based on a dual frequency technique. The significance of parasitic effects (including series resistance and the back metal contact of the metal-oxide-semiconductor (MOS) capacitor) on frequency dispersion was also studied. The effect of surface roughness on frequency dispersion is also discussed. After taking extrinsic frequency dispersion into account, the relaxation behavior can be modeled using the Curie-von Schweidler (CS) law, the Kohlrausch-Williams-Watts (KWW) relationship and the Havriliak-Negami (HN) relationship. Dielectric relaxation mechanisms are also discussed.

## 1. Introduction

With increasing demand for higher speed and device density, the device dimensions in Si complementary-metal-oxide-semiconductor (CMOS) based integration circuits are continually being scaled down, following what is termed as Moore’s law. The integrated circuit fabrication based on metal-oxide-semiconductor field-effect transistor (MOSFET) relies on thermally grown amorphous SiO_2_ as a gate dielectric [1,2,3]. However, according to the International Technology Roadmap for Semiconductors (ITRS), CMOS technology could be extended to 14 nm nodes by 2020 by adopting novel device structure and new materials. The physical gate length and printed gate length of the device can be scaled down to 6 nm and 9 nm, respectively [4]. The rapid shrinking of feature size of transistors has forced the gate channel length and gate dielectric thickness on an aggressive scale. As the thickness of SiO_2_ gate dielectric thin films used in metal-oxide-semiconductor (MOS) devices was reduced towards about 1 nm, the gate leakage current level became unacceptable. Below the physical thickness of 1.5 nm, the gate leakage current exceeds the specifications. To overcome this leakage problem, high-*k* materials were introduced because they allow the physical thickness of the gate stack to be increased but keep the equivalent oxide thickness (EOT) unchanged. Hence, the gate leakage was found to be reduced by two to three orders of magnitude.

On the other hand, capacitance-voltage (C-V) measurements are the fundamental characterization technique for MOS devices for the extraction of the oxide thickness [5], the maximal width of the depletion layer, interface trap densities [6], channel length [7], mobility [8], threshold voltage, bulk doping profile [9], and the distribution of the charges in dielectrics, which is used to evaluate the characterization of the interface states between the substrate and dielectric. Frequency dispersion in SiO_2_ has frequently been observed in C-V measurements [10,11]. Several models and analytical formulae have been thoroughly investigated for correcting the data from measurement errors. Attention has been given to eliminate the effects of series resistance [12], oxide leakage, undesired thin lossy interfacial layer between oxide and semiconductor [13], surface roughness [14], polysilicon depletion [15,16,17] and quantum mechanical effect [18,19,20,21].

In this paper, the extrinsic and intrinsic causes of frequency dispersion during C-V or *C*-*f* (capacitance-frequency) measurements in high-*k* thin films were investigated. In order to reconstruct the measured C-V curves for any given measurement data, parasitic components including imperfection of the back contact and silicon series resistance which was one of the extrinsic causes of frequency dispersion must be taken into account. The corrected capacitance was provided following related models. Furthermore, another extrinsic cause of frequency dispersion, lossy interfacial layer effect, on high-*k* MOS capacitances was investigated for zirconium oxides and then a four-element circuit model was introduced. On the other side, frequency dispersion from the effect of surface roughness was best demonstrated in ultra-thin SiO_2_ MOS devices [14] while the analysis of the La_x_Zr_1−x_O_2−δ_ thin film and Ce_x_Zr_1−x_O_2−δ_ thin film led to the conclusion that surface roughness was not responsible for the observed frequency dispersion for the thick high-*k* dielectric thin films. The polysilicon depletion effect and quantum confinement should be also considered. After taking into account all extrinsic causes of frequency dispersion mentioned above, the intrinsic effect (dielectric relaxation) of high-*k* dielectric thin films arose and several dielectric relaxation models were discussed. The dielectric relaxation results of Ce_x_Zr_1−x_O_2−δ_, LaAlO_3_, ZrO_2_ and La_x_Zr_1−x_O_2−δ_ thin films could be described by the Curie-von Schweidle (CS) law, the Kohlrausch-Williams-Watts (KWW) and the Havriliak-Negami (HN) relationship, respectively. The higher *k*-values were obtained from La_x_Zr_1-x_O_2-δ_ and Ce_x_Zr_1-x_O_2-δ_ thin films with the low lanthanide concentration levels (e.g., *x* ~ 0.1) where the more severe dielectric relaxation was observed. The causes of the dielectric relaxation were discussed in terms of this observation.

## 2. Experimental

**Figure 1:**
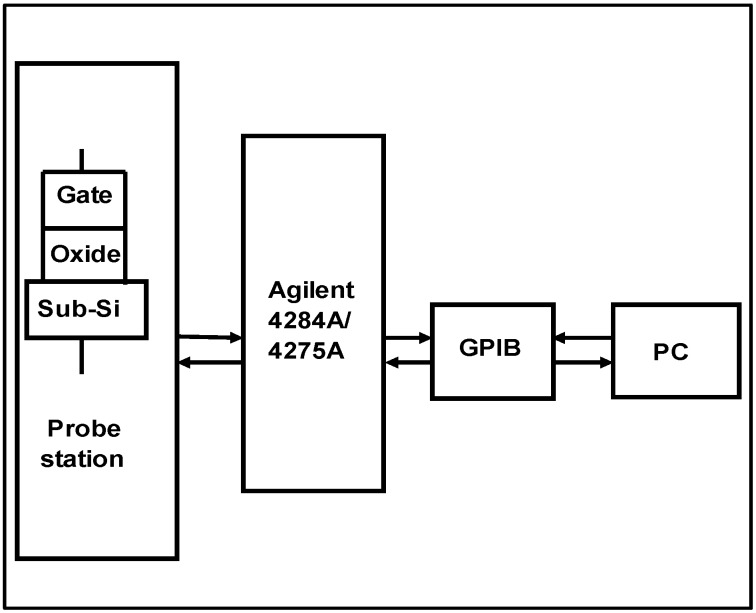
Capacitance-voltage (C-V) measurement system of metal-oxide-semiconductor (MOS) devices. A MOS device was located on the manual probe station which was connected to the LCR meters (Agilent 4284A/4275A). The LCR meters were controlled by a desktop computer through a GPIB interface. The C-V measurement data extracted from the LCR meters were transferred back to the computer and saved to obtain the C-V curves automatically.

The C-V and C-*f* measurements system consists of two Agilent precision LCR meters (4284A and 4275A), a desktop computer and a manual probe station. The MOS devices were wafer-probed on the probe station’s loading platform and were connected from Agilent 4284A/4275A to the desktop computer and the probe station together through a GPIB interface, as shown in Figure 1. The data measured from the LCR meters were transferred back to the computer and saved to obtain the C-V curves automatically.

**Figure 2:**
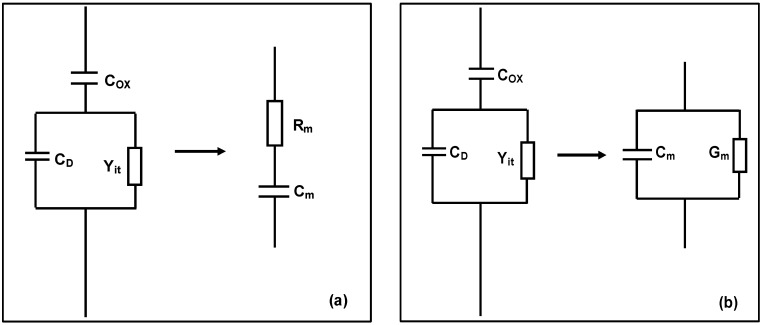
Conventional LCR meters typically measure the device capacitance based on (**a**) Series capacitance model or (**b**) Parallel capacitance model. *C_m_* is the measured capacitance. *R_m_* and *G_m_* are the measured resistance and conductance respectively. *C_D_* is the depletion capacitance and *Y_it_* is the admittance due to interface states of the MOS device, respectively.

The structure of the MOS device shown in Figure 1 is similar to planar capacitors which are formed by metal and dielectric. The differential capacitance of a MOS capacitor is:

(1) $$C=A\frac{dQ_{G}}{dV_{G}}=\frac{i_{ac}}{dV_{ac}/dt}$$

where *Q_G_* and *V_G_* are the charge area density and voltage on the metal electrodes, *A* is the metal electrode area, *dV_ac_/dt* is the AC voltage change, and *i_ac_* is the AC current. The capacitance of a MOS device was obtained by Agilent 4284A/4275A, which provided a small signal voltage variation rate (*dV_ac_/dt*) and measured the small signal current (*i_ac_*) flowing through the MOS device to calculate the differential capacitance of the MOS device according to Equation (1) [22,23]. For the Agilent 4284A/4275A precision LCR meters, there are two models used to calculate the device capacitance. One is the series model and the other is the parallel model, as shown in Figure 2. The parallel model was used in the following C-V and C-*f* measurements. In Figure 2, *C_m_* is the measured capacitance. *R_m_* and *G_m_* are the measured resistance and conductance respectively. *C_D_* is the depletion capacitance and *Y_it_* is the admittance due to interface states of the MOS device, respectively. *C_ox_* represents the actual frequency independent capacitance.

However, the influence of the leakage current of oxides to *i_ac_* in the C-V and C-*f* measurements of MOS devices by the LCR meters should be taken into account. Especially crystalline thin ﬁlms exhibit signiﬁcantly higher leakage current than amorphous thin ﬁlms, which could be due to the leakage pathway introduced from the grain boundaries and the local defects [24,25]. An approximation for the percentage instrumental error was given by the formula $0.1\times \sqrt{1+D^{2}}$, where *D* is a dissipation factor. If the instrumentation error is less than 0.3%, the leakage current in the MOS device is negligible [13]. In the following C-V and C-*f* measurements, the leakage current in high-*k* thin films was so small that it was not a contributing factor to frequency dispersion [26].

High*-k* dielectrics, LaAlO_3_, ZrO_2_, Ce_x_Hf_1−x_O_2−x_ and La_x_Zr_1−x_O_2−δ_ thin films, were deposited on n-type Si (100) substrates using liquid injection atomic layer deposition (ALD), carried out on an Aixtron AIX 200FE AVD reactor fitted with the “Trijet” ™ liquid injector system [27]. The doping level of Ce_x_Hf_1−x_O_2−x_ thin film and La_x_Zr_1−x_O_2−δ_ thin film was varied up to a concentration level of 63%, *i.e.*, *x* = 0.63. The interfacial layer between the high*-k* thin film and silicon substrate was a ~1 nm native SiO_2_ determined by cross-section transmission electron microscopy (XTEM). A thermal SiO_2_ sample was grown using dry oxidation at 1100 °C to provide a comparison with the high*-k* stacks. MOS capacitors were fabricated by thermal evaporation of Au gates through a shadow mask with an effective area of 4.9 × 10^−4^ cm^2^. The backside contact of selected Si wafers was cleaned with a buffer HF solution and subsequently a 200 nm thickness of Al film was deposited on it by thermal evaporation. Some selected samples of Ce_x_Hf_1−x_O_2−x_ thin films and La_x_Zr_1−x_O_2−δ_ thin films were annealed at 900 °C for 15 min in a N_2_ ambient to crystallize the thin films before metallization. All the other samples were annealed in forming gas at 400 °C for 30 min. The C-V or C-*f* curves of Ce_x_Hf_1−x_O_2−δ_, La_x_Zr_1−x_O_2−δ_, ZrO_2_, LaAlO_3_ and thermal SiO_2_ thin films were measured to investigate their electrical properties. X-ray diffraction (XRD), XTEM and atomic force microscopy (AFM) of La_x_Zr_1−x_O_2−δ_ thin films and Ce_x_Hf_1−x_O_2−δ_ thin films were used to investigate their physical properties.

## 3. Results and Discussion

Frequency dispersion was categorized into two parts: extrinsic causes and intrinsic causes. Section 3.1 presented the extrinsic frequency dispersion. After analyzing the C-V curves of SiO_2_ MOS capacitors (MOSC), the parasitic effect is introduced in Section 3.1.1. Dispersion could be avoided by depositing an Al thin film at the back of the silicon substrate. The correction models were able to minimize the dispersion as well. The existence of frequency dispersion in the LaAlO_3_ sample is discussed in Section 3.1.2, which is mainly due to the effect of the lossy interfacial layer between the high-*k* thin film and silicon substrate on the MOSC. Relative thicker thickness of the high-*k* thin film than the interfacial layer significantly prevented frequency dispersion. Also, extracted C-V curves were reconstructed by mathematic correction models. Frequency dispersion from the effect of surface roughness was represented in an ultra-thin SiO_2_ MOS device, which is discussed in Section 3.1.3. Furthermore, the surface property of the La_x_Zr_1−x_O_2−δ_ thin films is studied. In Section 3.1.4 two further potential extrinsic causes: polysilicon depletion effect and quantum mechanical confinement, for frequency dispersion are considered. After careful considerations of extrinsic causes for frequency dispersion, intrinsic frequency dispersion is analyzed in Section 3.2. Section 3.2.1 describes the frequency dependence of *k*-value in La_x_Zr_1−x_O_2_/SiO_2_ and Ce_x_Hf_1−x_O_2−δ_/SiO_2_ stacks. In order to interpret intrinsic frequency dispersion, several dielectric relaxation models are introduced in Section 3.2.2 for high-*k* materials with specified fitting parameters. Last but not least, three possible causes of the dielectric relaxation for the La_x_Zr_1−x_O_2−δ_ dielectrics are proposed in Section 3.2.3. The effects of the cation segregation caused by annealing and rapped electrons on the dielectric relaxation were negligible. However, a decrease in crystal grain size may be responsible for the increase in the dielectric relaxation.

### 3.1. Extrinsic Causes of Frequency Dispersion During C-V Measurement 

**Figure 3:**
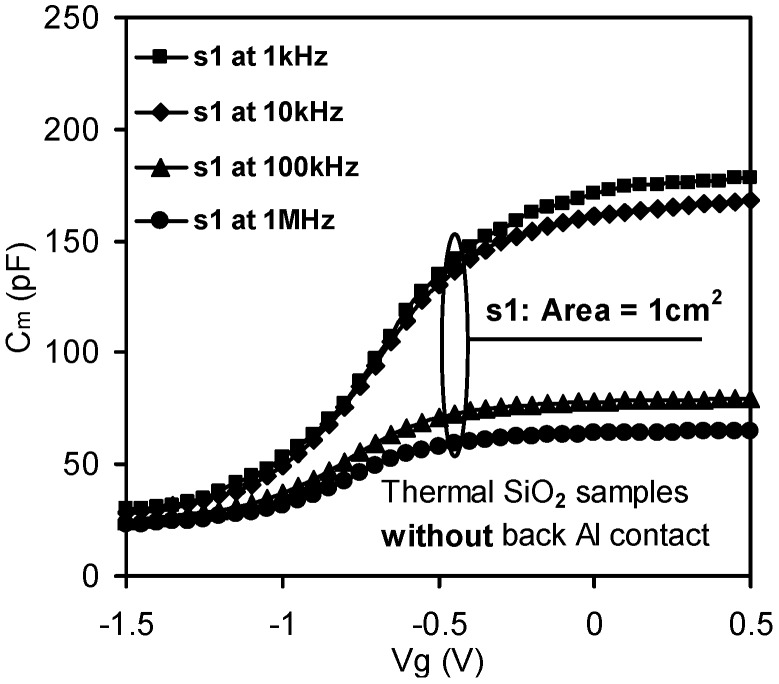
Frequency dispersion in C-V measurements observed in the thermal oxide (SiO_2_) sample. In the absence of a substrate back Al contact, dispersion was evident in the sample with a small substrate area of 1cm^2^ [32].

**Figure 4:**
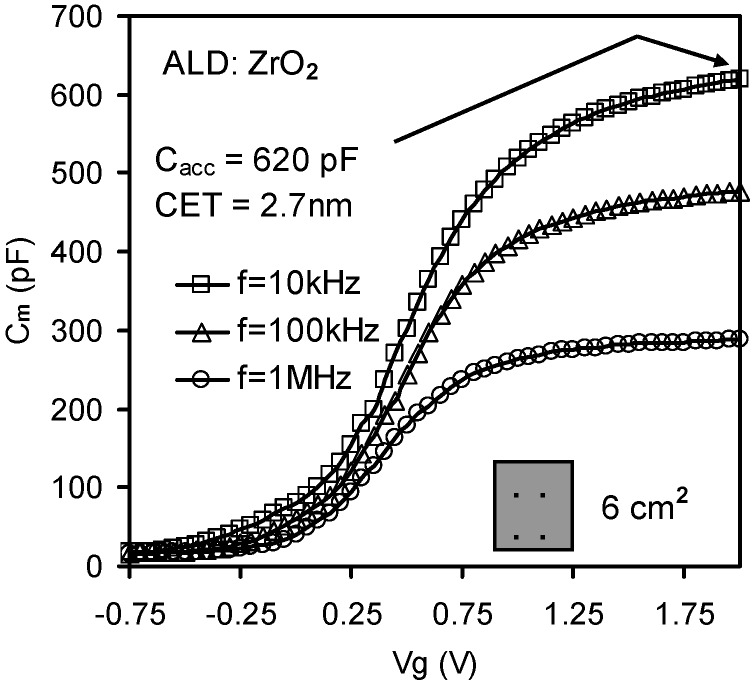
Presence of frequency dispersion in ZrO_2_ samples at different frequencies (10kHz, 100kHz and 1MHz). The shadowed boxes indicate the presence of metal Al contact at the back of silicon substrates with an effective area of 6 cm^2^ and the capacitance equivalent thickness (CET) is 2.7 nm. *C_acc_* is the capacitance in the accumulation range [32].

**Figure 5:**
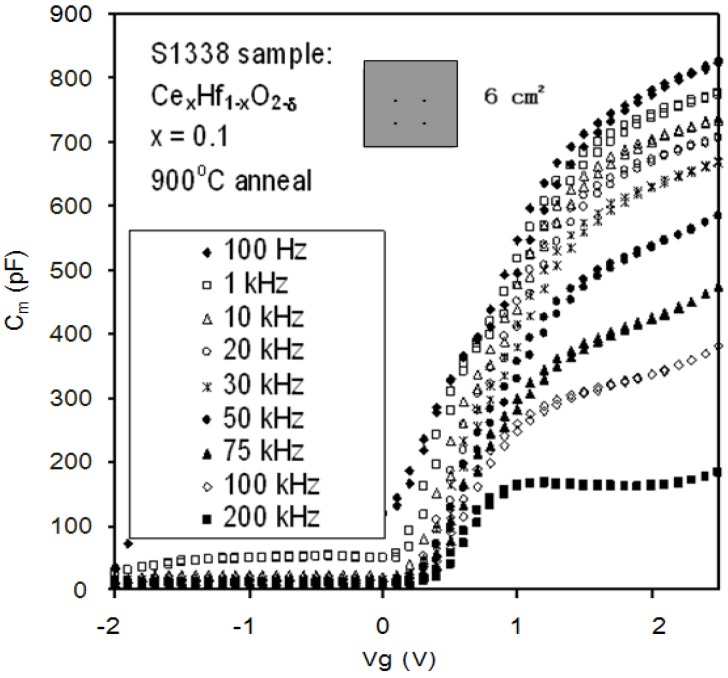
C-V curves from a Ce_x_Hf_1−x_O_2−δ_ thin film at different frequencies (from 100 Hz to 200 kHz). Frequency dispersion could still be observed regardless of the interfacial layer effect of MOS structures and parasitic effects (caused by substrate resistance, back contact imperfection and cables). This kind of dispersion was caused by the frequency dependence of the *k*-value (dielectric relaxation) [33].

**Figure 6:**
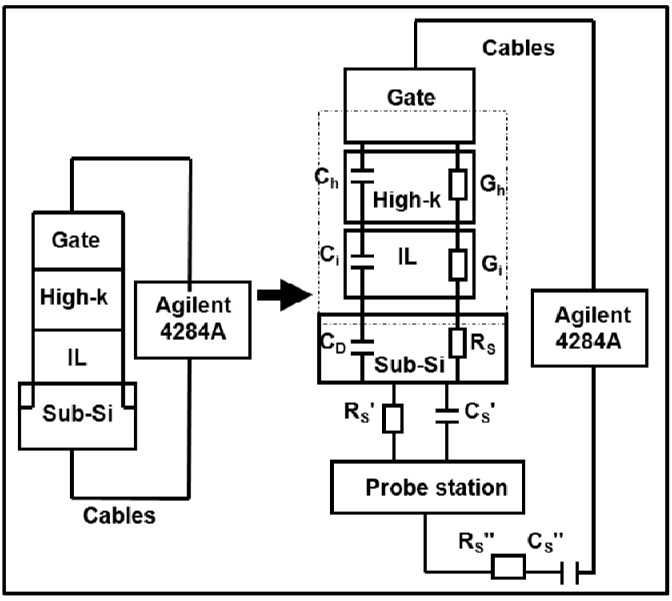
Causes of frequency dispersion during C-V measurement in the high-*k* thin film were the parasitic effect (including back contact imperfection resistance *R_S_^’^* and capacitance *C_S_^’^*, cables resistance *R_S_^”^* and capacitance *C_S_^”^*, substrate series resistance *R_S_* and depletion layer capacitance of silicon *C_D_*) and the lossy interfacial layer effect (interfacial layer capacitance *C_i_* and conductance *G_i_*). The dashed box includes surface roughness effect, polysilicon depletion effect, high-*k* capacitance *C_h_*, high-*k* conductance *G_h_*, the lossy interfacial layer capacitance *C_i_* and conductance *G_i_*. The oxide capacitance *C_ox_* consists of the high-*k* capacitance *C_h_* and the lossy interfacial layer capacitance *C_i_*.

Several reasons for unwanted frequency dispersions in SiO_2_ have been investigated, such as surface roughness [14], polysilicon depletion [15,16,17], quantum confinement (only for an ultra-thin oxide layer) [18,19,20,21], parasitic effect (including series resistance, back contact imperfection and cables connection) [28,29,30], oxide tunneling leakage current (direct tunneling current, F-N tunneling *etc*.) [31], unwanted interfacial lossy layer [13] and dielectric constant (*k*-value) dependence (dielectric relaxation) [26]. The extrinsic frequency dispersion is discussed firstly in Section 3.1. The extrinsic causes of frequency dispersion during C-V measurement in high-*k* thin film, which were investigated step by step before validating the effects of *k*-value dependence, were parasitic effect, surface roughness, and lossy interfacial layer. The other causes like tunneling leakage current and quantum confinement are negligible if the thickness of the high-*k* thin film is high enough. Polysilicon depletion effects were not considered due to the fact that metal gates were used here. The C-V results of high-*k* or SiO_2_ based dielectrics are shown in Figure 3, Figure 4 and Figure 5, respectively. The parasitic effect (including back contact imperfection *R_S_^’^*, *C_S_^’^*, cables *R_S_^”^*, *C_S_^”^* and substrate resistance R_S_), the lossy interfacial layer effect *C_i_*, *G_i_* (between the high-*k* thin film and silicon substrate), polysilicon depletion effect and surface roughness on high-*k* thin films are summarized in detail in Figure 6.

#### 3.1.1. Parasitic Effect

Parasitic effects in MOS devices included parasitic resistances and capacitances such as bulk series resistances, series contact, cables and many other parasitic effects [34]. Five different sources of parasitic series resistance have been suggested [35]. However, only two of them which have practical importance are listed as follows: (1) the series resistance *R_S_* of the quasi-neutral silicon bulk between the back contact and the depletion layer edge at the silicon surface underneath the gate; and (2) the imperfect contact of the back of the silicon wafer. Frequency dispersion caused by the parasitic effect is shown in Figure 3.

**Figure 7:**
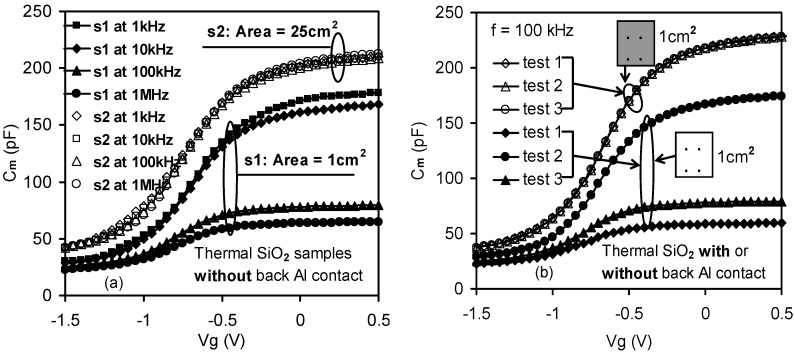
Frequency dispersion in C-V measurements observed in thermal oxide (SiO_2_) samples. (**a**) In the absence of substrate back Al contact, dispersion was evident only in the sample with a smaller substrate area (denoted by s1); (**b**) The reproducibility of the tested devices in both the presence and absence of back metal contact. Both of the sample sets were measured three times within 24 hours. Closed symbols (e.g., ▲) signified the C-V results from the sample without back Al contact (indicated by a blank square), while the opened symbols (e.g., ○) showed the C-V results from the other sample with back Al contact (indicated by a shadow square) [32].

The significance of the series resistance effect, which was commonly due to silicon bulk resistance and back contact imperfection, was best demonstrated in thermal SiO_2_ MOS capacitors, since in this case the effect of the lossy interfacial layer between the bulk dielectric and silicon substrate can be neglected. The thickness of thermal SiO_2_ was thick enough to allow the tunneling leakage current to be neglected. [36,37]. Frequency dispersion in the SiO_2_ capacitor was only observed in samples with small substrate effective areas as depicted in Figure 7a (closed symbols extracted from Figure 3). In addition, the measured results were also no longer reproducible for small samples in the absence of Al back contacts, as shown in Figure 7b (the closed symbols). It therefore impacted the measurement reliability.

**Figure 8:**
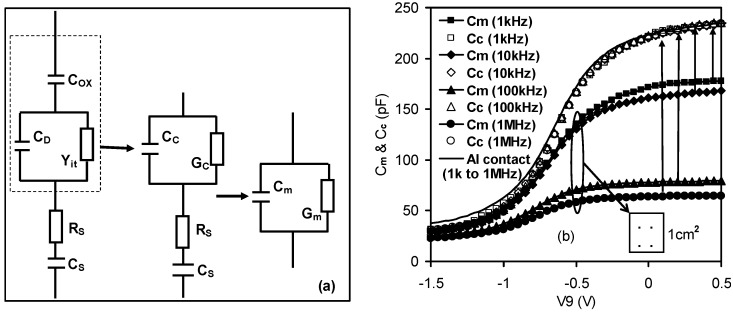
Effects of series resistance and back contact imperfection. (**a**) Equivalent circuit model, taking into account the presence of parasitic components from series resistance, cables and back contact imperfection (with the addition of the *C_S_* and *R_S_*). *C_D_* is the depletion capacitance of silicon and Y_it_ is the admittance due to the interface states between SiO_2_ and silicon substrate, respectively. *C_ox_* is the oxide capacitance; (**b**) Extracted C_C_-Vg curves based on measured data *C_m_* and *G_m_* using Equation (2). Dispersions disappear after considering *C_S_* and *R_S_* or depositing back Al contact (solid line). The blank square shows the tested device without back Al contact on silicon substrate. The effective substrate area is 1cm^2^ [32].

In order to reconstruct the measured C-V curves for any given measurement data in the frequency domain for SiO_2_, one must take into account the parasitic components that may arise due to the silicon series resistance and the imperfection of the back contact. A correction may then be applied for the measured C-V curves in order to obtain their true values. Figure 8a shows an equivalent circuit of an actual case in comparison with the measurement mode, where *C_ox_* represents the actual frequency independent capacitance across the SiO_2_ gate dielectric, *R_S_* includes both the bulk resistance in the silicon substrate and contributions from various contact resistances and cable resistances. The presence of the back contact capacitance and contributions from cable capacitance were also modeled by a capacitance. *C_S_*, *C_C_*, *G_C_*, *C_m_*, *G_m_* refer to corrected (without the effect of the parasitic components *R_S_* and *C_S_*) measured capacitance and conductance, respectively. Following Kwa [13], the corrected capacitance *C_C_* was given by [32]:

(2) $$C_{C}\;=\;\frac{\left(\omega^{2}C_{m}C_{p}-G_{m}^{2}-\omega^{2}C_{m}^{2}\right)\left(G_{m}^{2}+\omega^{2}C_{m}^{2}\right)C_{p}}{\omega^{2}C_{p}^{2}{\left[G_{m}\left(1-G_{m}R_{S}\right)-\omega^{2}C_{m}^{2}R_{S}\right]}^{2}+{\left(\omega^{2}C_{m}C_{p}-G_{m}^{2}-\omega^{2}C_{m}^{2}\right)}^{2}}$$

(3) $$C_{p}=\frac{C_{ox}(G_{ma}^{2}+\omega^{2}C_{ma}^{2})}{\omega^{2}(C_{ma}^{2}C_{ox}-C_{ma}^{2})-G_{ma}^{2}}$$

(4) $$R_{S}=\frac{G_{ma}}{G_{ma}^{2}+\omega^{2}C_{ma}^{2}}$$

where *C_ma_* and *G_ma_* are the capacitance and conductance measured in strong accumulation. The measured capacitance can be recovered, independently of the measured frequencies, by applying the correction according to the model as depicted in Figure 8b. Alternatively, the parasitic effects can simply be minimized by depositing an Al thin film at the back of the silicon substrate (open symbols in Figure 7b and solid line in Figure 8b). In summary, it has been demonstrated that once the parasitic components are taken into account, it is possible to determine the true capacitance values free from errors. Therefore, the measurement system reliability can be maintained.

#### 3.1.2. Lossy Interfacial Layer Effect

**Figure 9:**
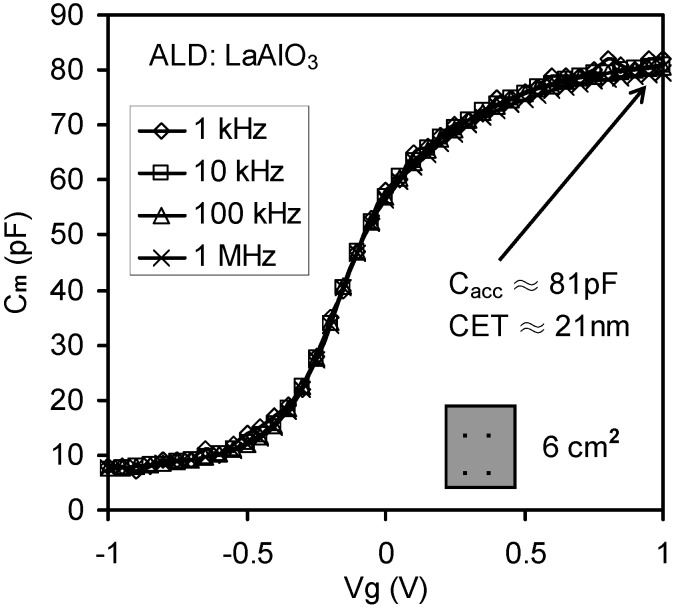
High frequency C-V results of LaAlO_3_ thin film. The absence of frequency dispersion in the LaAlO_3_ sample is observed with an effective area of 6 cm^2^ with back Al contact. *C_acc_* is the capacitance in the accumulation range [32].

Concerning Figure 4, it should be noted that the dispersion was not caused by parasitic effects, since this sample had a large substrate area and an Al thin film was deposited on the back of the wafer. Subsequently, the effect of the lossy interfacial layer between the high-*k* thin film and silicon substrate on the high*-k* MOSC was investigated. The absence of frequency dispersion observed in Figure 9 may be explained in terms of the relative thickness of the high-*k* thin film compared to the interfacial layer. For the sample for Figure 9 the interfacial layer thickness (~1 nm) was negligible compared with the capacitance equivalent thickness (CET) of ~ 21 nm. Therefore in this case the high-*k* layer capacitance was much less than the interfacial layer capacitance (*i.e.*, *C_h_* << *C_i_*) and the effect of *C_i_* on *C_m_* was eliminated. Furthermore the effect of the lossy interfacial layer conductance *G_i_* on frequency dispersion can be suppressed by replacing the native SiO_2_ by a denser SiO_2_ thin film. In Figure 4, the frequency dispersion effect was significant even with the Al back contact and the bigger substrate area. In this case, *C_h_* (CET = 2.7 nm) was comparable with *C_i_* (~1 nm native SiO_2_) and the frequency dispersion effect was attributed to losses in the interfacial layer capacitance, caused by interfacial dislocation and intrinsic differences in bonding coordination across the chemically abrupt ZrO_2_/SiO_2_ interface.

**Figure 10:**
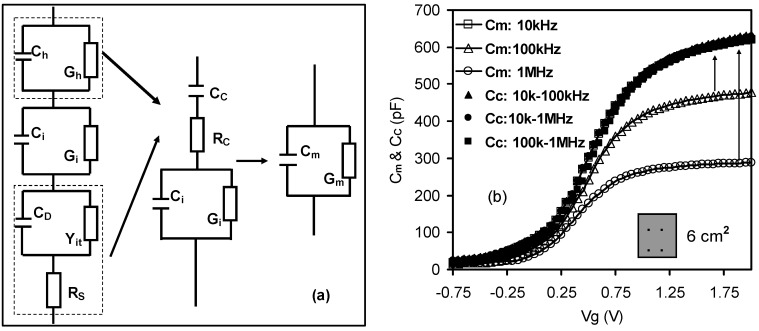
Effect of the lossy interfacial layer on high*-k* stacks. (**a**) Four-element equivalent circuit model for high*-k* stacks, taking into account the presence of the interfacial layer with the additional capacitance, *C_i_*, and conductance, *G_i_*, parallel circuit components. *C_h_* and *G_h_* represent the actual capacitance and conductance across the high*-k* dielectric. *C_D_* is the depletion capacitance and *Y_it_* is the admittance due to interface states, respectively; (**b**) Extracted C_C_-Vg curves based on dual-frequency data from Figure 3 and the equivalent circuit model from Figure 10a [32].

Based on the above explanation, Figure 10a showed a four-element circuit model for high-*k* stacks, adapted from a dual frequency technique [10], with the capacitance value reconstructed from the loss. The expression for the corrected capacitance, *C_C_*, was [32]:

(5) $$C_{C}=\frac{{\text{Δ}}^{2}\left(\omega_{1}^{2}-\omega_{2}^{2}\right)}{\left[I_{m2}\omega_{2}\omega_{1}^{2}\left({\text{Δ}}^{2}+\omega_{2}^{2}\right)-I_{m1}\omega_{1}\omega_{2}^{2}\left({\text{Δ}}^{2}+\omega_{1}^{2}\right)\right]}\;\text{Δ}=\frac{\omega_{1}I_{m1}-\omega_{2}I_{m2}}{R_{m1}-R_{m1}},\;I_{mj}=\frac{\omega_{j}C_{mj}}{\left(G_{mj}^{2}+\omega_{j}^{2}C_{mj}^{2}\right)},\;R_{mj}=\frac{G_{mj}}{\left(G_{mj}^{2}+\omega_{j}^{2}C_{mj}^{2}\right)}\;and\;j=1,2.$$

where *C_m_* and *G_m_* are the measured capacitance and conductance and *ω* is the measurement angular frequency. At an angular frequency *ω_j_* (*j* = 1 or 2), the measured capacitance and conductance are *C_mj_* and *G_mj_* respectively. Since the expression of *C_C_* with respect to *ω_j_*, *C_mj_* and *G_mj_* is complicated, three abstract parameters, *Δ*, *I_mj_* , and *R_mj_* have been introduced to reduce the expression of *C_C_*. Figure 10b shows the corrected C-V curves from Figure 4, extracted using Equation (5). All of the extracted C-V curves closely align with one another over the three different frequency pairs to reconstruct the true capacitance values. This indicates that the presence of a lossy interfacial layer is also responsible for the effect of frequency dispersion in high-*k* stacks.

#### 3.1.3. Surface Roughness Effect

**Figure 11:**
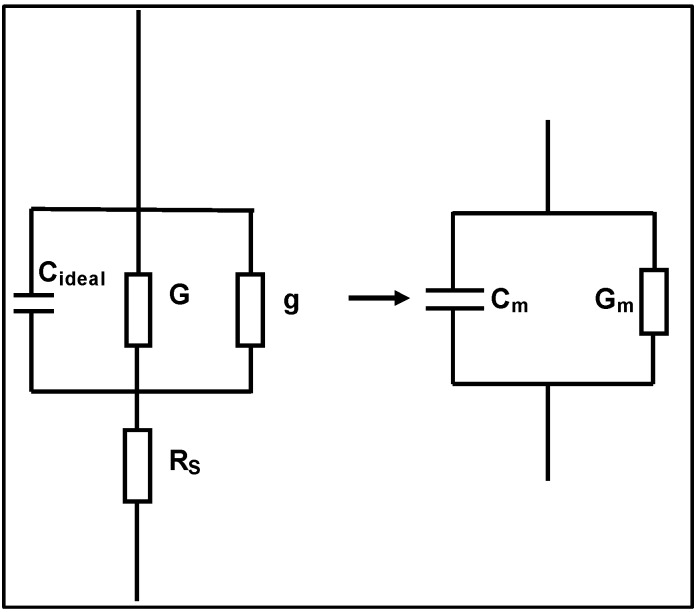
Equivalent circuit of the parallel mode of the measurement system. *G* is the conductance due to pure tunneling effect. *g* is the conductance due to the surface roughness effect. *R_S_* is the series resistance. Figure is taken from Reference 36.

**Figure 12:**
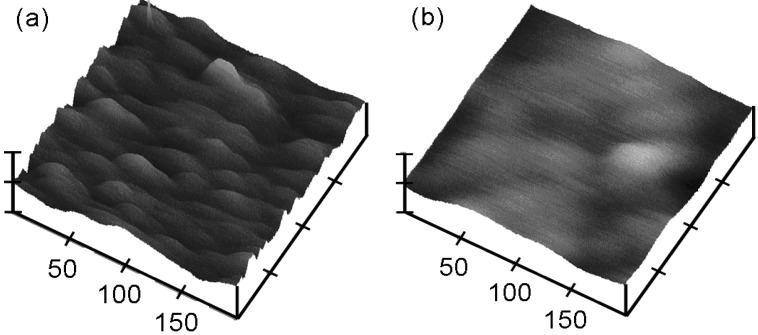
AFM micrographs of the surface of La_x_Zr_1−x_O_2_ annealed thin films. (**a**) *x* = 0.35; (**b**) *x =* 0.09 [33].

After taking the parasitic effects and the lossy interfacial layer effect into account, the unwanted frequency dispersion shown in Figure 5 may be caused by surface roughness. Frequency dispersion from the effect of surface roughness is best demonstrated in an ultra-thin SiO_2_ MOS device [14]. In the following discussion, the effects of direct tunneling, series resistance and surface roughness on the capacitance were taken into account without considering quantum confinement and the polysilicon depletion effect. From Figure 11, the measured capacitance *C_m_* is given by [38]:

(6) $$C_{m}=\frac{C_{ideal}}{{\left[(G+g)R_{S}+(\omega C_{ideal}R_{S})\right]}^{2}}$$

where *G* is the conductance due to a pure tunneling effect, *g* is the conductance due to the surface roughness effect, and *R_S_* is the series resistance. From Equation (6), the real capacitance taking into account the surface roughness, *C_ideal_*, can be calculated and it is free of frequency [38]. It was found that the surface roughness affects frequency dispersion when the thickness of ultra-thin oxides is ~1.3nm. To investigate whether the unwanted frequency dispersion of the high-*k* materials in Figure 5 is caused by the surface roughness or not, the surface properties of the La_x_Zr_1−x_O_2−δ_ thin films was studied using AFM. The typical AFM micrographs of the La_x_Zr_1−x_O_2−δ_ annealed thin films (*x* = 0.35 and *x =* 0.09) are shown in Figure 12.

**Figure 13:**
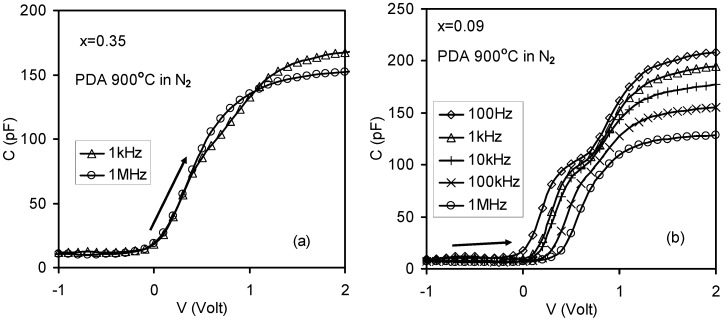
C-V results at different frequencies from the annealed La_x_Zr_1−x_O_2−δ_ samples after back Al contact deposition and the effective substrate area was 6 cm^2^: (**a**) *x* = 0.35; and (**b**) *x* = 0.09. Signiﬁcant frequency dispersion was observed for the *x* = 0.09 annealed sample, but not for the *x* = 0.35 annealed sample [26].

The Root Mean Square (RMS) roughness of the *x* = 0.35 thin film is 0.64 nm after annealing, as shown in Figure 12a. However no significant roughness was observed for the *x* = 0.09 thin film (RMS roughness of 0.3 nm), as shown in Figure 12b [26]. It means that the *x* = 0.35 thin film has more surface roughness than the *x* = 0.09 thin film. The applied frequency increased from 1 kHz to 1 MHz. The annealed thin film with *x* = 0.09 had large frequency dispersion where the capacitance decreased from 192 pF to 123 pF and the frequency changed from 1 kHz to 1 MHz. However, the annealed thin film with *x* = 0.35 showed small frequency dispersion where the capacitance decreased from 167 pF to 151 pF and the frequencies changed from 1kHz to 1MHz. Comparing these results from the C-V measurements in Figure 13, it leads to the conclusion that the surface roughness is not responsible for the observed frequency dispersion of the high-*k* dielectric thin films in Figure 13.

#### 3.1.4. Other Effects

There were two further potential causes of the observed frequency dispersion, polysilicon depletion effect and quantum mechanical confinement, for frequency dispersion which are not important for the samples in this paper. For thinner oxides, the poly depletion effect will become more significant leading to reduced surface potential, channel current, and gate capacitance. Furthermore, poly depletion will affect the extraction of the physical oxide thickness [39,40,41,42]. Some analysis and numerical results for the polysilicon depletion effect on the MOS device have been proposed [43,44]. The decrease in the gate capacitance caused by polysilicon depletion can be assumed as a cause of the increase in the effective gate oxide thickness. There are many surface potential models, which can be used to analyze the gate capacitance, solved by the Poisson Equation with boundary conditions to investigate the polysilicon depletion effect [45]. However, the polysilicon depletion effect was not under consideration for the samples used in this paper because the gates of the MOS capacitor samples were metal (Al or Au) fabricated by thermal evaporation through a shadow mask.

For oxide thicknesses down to 1~3 nm, the quantum mechanical effect should be taken into account [46,47,48]. There was a difference between the calculated capacitance and the measured capacitance with ultra-thin gate dielectrics. Quantum mechanical confinement would result in the continuous band being quantized into electric sub-band near the surface. The additional band bending confines the carriers to the narrow surface channel. The electron position changes and the peak of electron density is no longer in the silicon/silicon oxide interface, which would be further away from the surface in MOS devices [49,50]. However since the thickness of the high-*k* layer and interfacial layer is greater than 3 nm in the samples considered for this paper, the quantum mechanical effects were not considered.

### 3.2. Intrinsic Causes of Frequency Dispersion During C-V Measurements

#### 3.2.1. Frequency Dependence of *k*-Value

**Figure 14:**
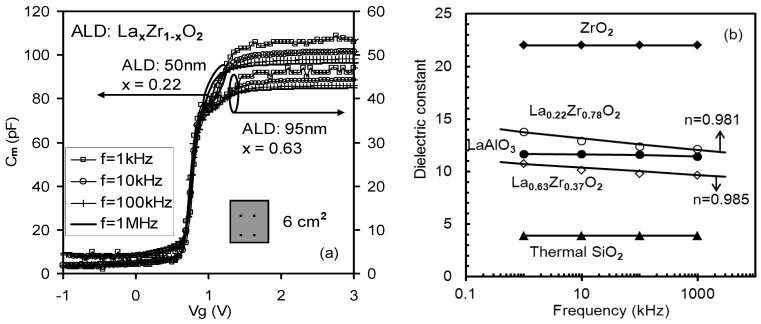
(**a**) Frequency dispersion in C-V measurements observed from La_x_Zr_1−x_O_2_ samples after back Al contact deposition and the effective substrate area was 6 cm^2^. Therefore, all the extrinsic causes of frequency dispersion were excluded; (**b**) A summary of frequency dependence of *k*-value extracted from Figure 14a, Figure 7 (SiO_2_), Figure 9 (LaAlO_3_), and Figure 10 (ZrO_2_). No frequency dependence of *k*-value was observed for the LaAlO_3_/SiO_2_ and ZrO_2_/SiO_2_ stacks. The frequency dependence of the *k*-value was observed for the La_x_Zr_1−x_O_2_/SiO_2_ stacks [32].

**Figure 15:**
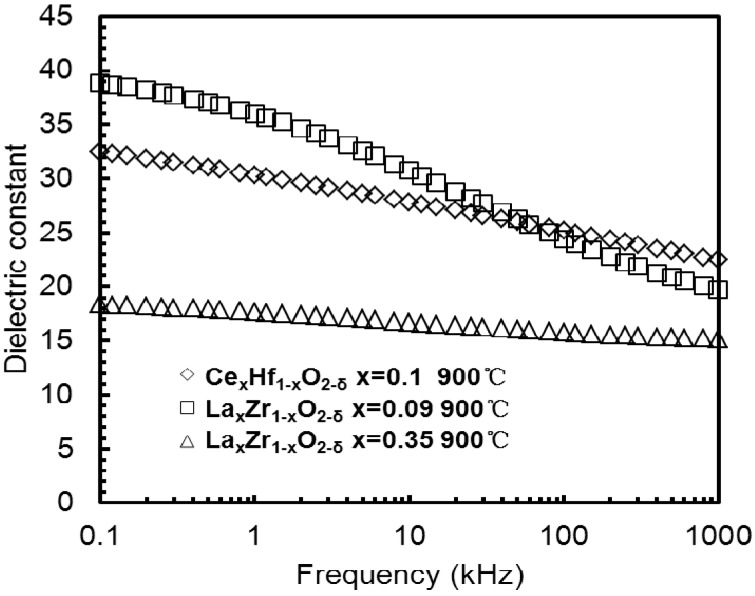
Frequency dependence of the *k*-value was extracted from C-*f* measurements of La_0.35_Zr_0.65_O_2−δ_ and La_0.09_Zr_0.91_O_2−δ_ thin films annealed at 900 °C, or extracted from Figure 13 (a,b). Frequency dependence of the Ce_x_Hf_1−x_O_2−δ_ thin film was extracted from Figure 5 [33].

**Figure 16:**
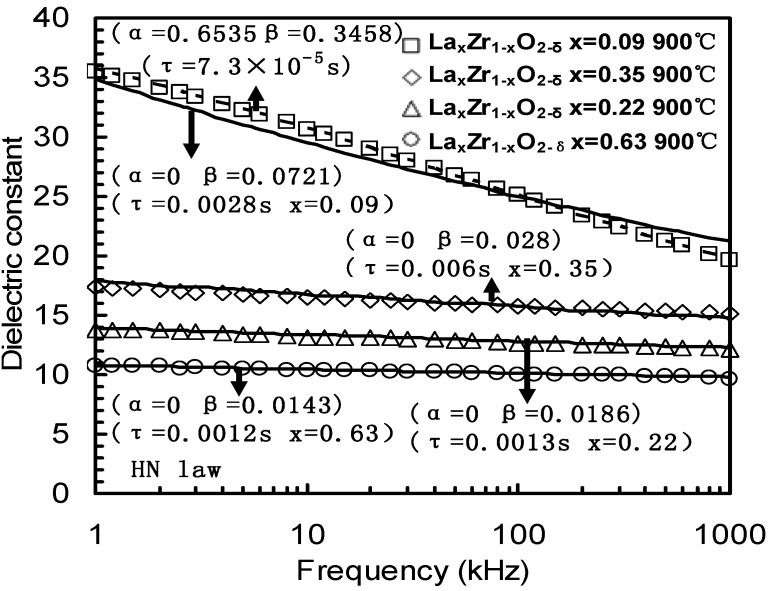
Frequency dependence of the *k*-value was extracted from C-*f* measurements observed in four La_x_Zr_1−x_O_2−δ_ thin films. The square-symbols were measured from the La_0.09_Zr_0.91_O_2−δ_ sample. The diamond-symbols were measured from the La_0.35_Zr_0.65_O_2−δ_ sample. The triangle-symbols were measured from the La_0.22_Zr_0.78_O_2−δ_ sample. The circle-symbols were measured from the La_0.63_Zr_0.27_O_2−δ_ sample. Solid lines are from fitting results from the Cole-Davidson equation, while the dashed line is from the HN equation. The parameters *α*, *β* and *τ* are parameters from the Cole-Davidson or HN equation [32,33].

Extrinsic causes of frequency dispersion during C-V measurements in high-*k* materials have been taken into account. Frequency dispersion can now solely be associated with the frequency dependence of the *k*-value in Figure 5, Figure 13 and Figure 14a. The frequency dependence of the *k*-value can be extracted as shown in Figure 14b, Figure 15 and Figure 16. The details are given below.

C-V data from the annealed thin films (La_0.22_Zr_0.78_O_2_ and La_0.63_Zr_0.37_O_2_) are given in Figure 14a. Figure 14b showed no frequency dependence of the *k*-value in LaAlO_3_/SiO_2_ and ZrO_2_/SiO_2_ stacks. However, the frequency dependence of the *k*-value was observed in La_x_Zr_1__–x_O_2_/SiO_2_ stacks. The *k*-values of La_0.22_Zr_0.78_O_2_ and La_0.63_Zr_0.37_O_2_ were observed and separately decreased from 13.5, 10.5 to 12 and 9.5 as the frequency increased from 1 kHz to 1 MHz. A constant frequency response was observed in thermal SiO_2_, as shown in Figure 14b.

The *k*-*f* (*k*-value-frequency) data of the Ce_x_Hf_1−x_O_2−δ_, La_0.35_Zr_0.65_O_2−δ_ and La_0.09_Zr_0.91_O_2−δ_ thin films are given in Figure 15. The zirconia thin film with a lanthanum (La) concentration of *x* = 0.35 showed that a *k*-value slowly decreased from 18 to 15 as the frequency increased from 100 Hz to 1 MHz. In contrast the lightly doped 9% sample had a sharp decreased *k*-value and suffered from a severe dielectric relaxation. A *k*-value of 39 was obtained at 100Hz, but this value was reduced to a *k*-value of 19 at 1 MHz. The 10% Ce doped hafnium thin film also had a *k*-value change from 33 at 100 Hz to 21 at 1 MHz. Figure 16 summarizes the frequency dependence of *k*-value of four La_x_Zr_1–x_O_2_ thin films from Figure 14 and Figure 15.

Many dielectric relaxation models were proposed to interpret intrinsic frequency dispersion which is also termed as frequency dependence of *k*-value. The fitted parameters of the dielectric relaxation models for Figure 16 are shown in Table 1. The details of the models are discussed below.

**Table 1:** The fitted parameters of the dielectric relaxation models for Figure 16.

Models	Cole-Cole		Cole-Davidson		Havriliak-Negami		
Parameters	*α*	*τ* (s)	*β*	*τ* (s)	*α*	*β*	*τ* (s)
La_x_Zr_1–x_O_2–δ_	0.75	3.9 × 10^−7^	0.0721	0.0028	0.6535	0.3458	7.3 × 10^−5^
*x*=0.09							
La_x_Zr_1–x_O_2–δ_	0.866	4.6 × 10^−11^	0.028	0.006	0	0.028	0.006
*x*=0.35							
La_x_Zr_1–x_O_2–δ_	0.815	3.8 × 10^−11^	0.0186	0.0013	0	0.0186	0.0013
*x*=0.22							
La_x_Zr_1–x_O_2–δ_	0.82	5.2 × 10^−12^	0.0143	0.0012	0	0.0143	0.0012
*x*=0.63							

#### 3.2.2. Dielectric Relaxation Models and Data Fitting

In 1929, Debye [51] described a model for the response of electric dipoles in an alternating electric field. This model led to a description for the complex dielectric constant ε*. The Debye equation and its real part are [51]:

(7) $$\varepsilon^{*}(\omega )=\varepsilon_{\infty }+(\varepsilon_{s}-\varepsilon_{\infty })/\left[1+(i\omega \tau \right)]$$

(8) $$\varepsilon^{\prime }(\omega )=\varepsilon_{\infty }+(\varepsilon_{s}-\varepsilon_{\infty })/[1+\omega^{2}\tau^{2}]$$

where *τ* is called the relaxation time which is a function of temperature and it is independent of the time, angular frequency *ω* = 2π*f*. At static conditions the dielectric behavior is characterized by the relative static dielectric permittivity *ε_s_*, which is usually denoted as “static dielectric constant”. *ε_s_* is also deﬁned as the zero-frequency limit of the real part, *ε'*, of the complex permittivity. *ε_∞_* is the dielectric constant at ultra-high frequency. *ε'* is the *k*-value.

The Debye theory assumed that the molecules are spherical in shape and dipoles are independent in their response to the alternating field with only one relaxation time. The Debye equation (8) predicates *ε'* sharp decreases with frequency over a relatively small band width. Generally, the Debye theory of dielectric relaxation is utilized for particular type polar gases, dilute solutions of polar liquids and polar solids. [52] However, the dipoles for a majority of materials are more likely to be interactive and dependent in their response to the alternating field. Therefore, very few materials completely agree with the Debye equation which has only one relaxation time. The Debye expression cannot interpret the data of polar dielectrics with a distribution of relaxation times (comparing to one relaxation time) [53]. For example, Figure 15 shows that the intrinsic frequency dispersion of the high-*k* materials (La_x_Zr_1–x_O_2_ and Ce_x_Hf_1–x_O_2–δ_) occurred over a wide frequency range. The data was unable to be fitted with the Debye equation because the high-*k* materials have more than one relaxation time.

Since the Debye expression cannot properly predict the behavior of some liquids and solids such as chlorinated diphenyl at −25 °C and cyclohexanone at −70 °C [52], Cole K.S. and Cole R.H. proposed an improved Debye equation, known as the Cole-Cole equation, to interpret data observed on various dielectrics. Among relaxation frequencies Cole-Cole relaxation showed that *ε'* decreased more slowly with frequency than the Debye relaxation. By observing a large number of materials, they found that when the imaginary part (*ε''*) was plotted versus *ε'*, a curved arc resulted, whereas a semicircle was predicted by the Debye relation. The Cole-Cole equation can be represented by ε*(*ω*) [52]:

(9) $$\varepsilon *(\omega )=\varepsilon_{\infty }+(\varepsilon_{s}-\varepsilon_{\infty })/[1+{\left(i\omega \tau \right)}^{1-\alpha }]$$

where *τ* is relaxation time and *α* is a constant for a given material, having a value 0 ≤ *α* ≤ 1. *α* = 0 for Debye relaxation. The real part of the Cole-Cole equation is:

(10) $$\varepsilon^{\prime }(\omega )=\varepsilon_{\infty }+(\varepsilon_{s}-\varepsilon_{\infty })\frac{1+{\left(\omega \tau \right)}^{1-\alpha }\text{sin}\frac{1}{2}\alpha \pi }{1+2{\left(\omega \tau \right)}^{1-\alpha }\text{sin}\frac{1}{2}\alpha \pi +{\left(\omega \tau \right)}^{2(1-\alpha )}}$$

The larger the value of *α*, the larger is the distribution of relaxation times. The Cole-Cole equation can be used to fit the dielectric relaxation results shown in Figure 16 of the La_0.91_Zr_0.09_O_2_, La_0.22_Zr_0.78_O_2_, La_0.35_Zr_0.65_O_2_ and La_0.63_Zr_0.37_O_2_ thin films and the fitted parameters are shown in Table 1. All of the data perfectly fitted, but the relaxation time was too small (e.g., 10^−11^s), as shown in Table 1.

Davidson *et al.* [53] proposed the following expression (Cole-Davidson equation) to interpret data observed on propylene glycol and glycerol based on the Debye expression:

(11) $$\varepsilon *(\omega )=\varepsilon_{\infty }+(\varepsilon_{s}-\varepsilon_{\infty })/{\left(1+i\omega \tau \right)}^{\beta }$$

where *τ* is the relaxation time and *β* is a constant for a given material. 0 ≤ *β ≤* 1 which controlled the width of the distribution and *β* = 1 for Debye relaxation. The smaller the value of *β* then the larger is the distribution of relaxation times [54]. For angular frequencies *ω* > 1/τ, the Cole-Davidson model exhibits an asymmetric broadening of the spectrum towards high frequency. The data of propylene glycol and glycerol can be fitted with the Debye formula in a low frequency region. However, at high frequencies, the Debye formula is no longer suitable for fitting. The data can be properly fitted by the Cole-Davidson formula instead. [55] This was reported as a limiting case to the Debye equation. The asymmetric loss factor *ε''* was more seriously in error as the parameter *β* increased.

The real part of Equation (11) is given by [56]:

(12) $$\varepsilon^{\prime }(\omega )=\varepsilon_{\infty }+(\varepsilon_{s}-\varepsilon_{\infty }){\left(\text{cos}\phi \right)}^{\beta }\text{cos}\beta \phi$$

(13) ϕ=arctg(ωτ)

The Cole-Davidson equation could also fit the dielectric relaxation results shown in Figure 16 and the fitted parameters are shown in Table 1. However, the fitting for the La_0.91_Zr_0.09_O_2_ thin films was not acceptable.

Both the Cole-Cole and Cole-Davidson equations are empirical and could be considered to be the consequence of the existence of a distribution of relaxation times rather than that of the single relaxation time (Debye equation). The physical reason for the distribution of relaxation times in the Cole-Cole and Cole-Davidson empirical equations is not yet clear. The reason for a distribution of relaxation times has been made in certain particular cases, e.g., the occurrence of protonic resonance (reported by Kliem and Arlt [57]) and the porosity effect (proposed by Cabeza *et al*. [58]).

In 1966, S. Havriliak and S. J. Negami reported the Havriliak-Negami (HN) equation which combined Cole-Cole and Cole-Davidson equations for twenty one polymers [59,60]. The HN equation is [60]:

(14) $$\varepsilon *(\omega )=\varepsilon_{\infty }+(\varepsilon_{s}-\varepsilon_{\infty })/{\left(1+{\left(i\omega \tau \right)}^{1-\alpha }\right)}^{\beta }$$

The real part of the HN equation is given by [61]:

(15) $$\varepsilon^{\prime }(\omega )=\varepsilon_{\infty }+(\varepsilon_{s}-\varepsilon_{\infty })\frac{\text{cos}(\beta \varphi )}{{\left(1+2{\left(\omega \tau \right)}^{1-\alpha }\text{sin}(\pi \alpha /2)+{\left(\omega \tau \right)}^{2(1-\alpha )}\right)}^{\beta /2}}$$

(16) $$\varphi =arctg\frac{{\left(\omega \tau \right)}^{1-\alpha }\text{cos}\frac{1}{2}\pi \alpha }{1+{\left(\omega \tau \right)}^{1-\alpha }\text{sin}\frac{1}{2}\pi \alpha }$$

where *α* and *β* are the two adjustable ﬁtting parameters. *α* is related to the width of the loss peak and *β* controls the asymmetry of the loss peak [62]. In this model, parameters *α* and *β* could both vary between 0 and 1. The Debye dielectric relaxation model with a single relaxation time from Equation (14) *α* = 0 and *β* = 1, the Cole-Cole model with symmetric distribution of relaxation times follows for *β* = 1 and 0 ≤ *α* ≤ 1, and the Cole-Davidson model with an asymmetric distribution of relaxation times follows for *α* = 0 and 0 ≤ *β* ≤ 1. The HN equation had two distribution parameters *α* and *β* but Cole-Cole and Cole-Davidson equations had only one.

This relaxation function had two intriguing features associated with it. First, and most importantly, it represented the experimental quantities almost within their reliability. Secondly, this function could be considered as a generalized way of writing the two known and well documented dispersion functions of Cole [52]. Hartmann *et al.* [62] have shown that the five parameters HN model used in the frequency domain can accurately describe the dynamic mechanical behavior of polymers, including the height, width, position, and shape of the loss peak.

The HN equation can be fitted for the dielectric relaxation results of the four La_0.91_Zr_0.09_O_2_, La_0.22_Zr_0.78_O_2_, La_0.35_Zr_0.65_O_2_ and La_0.63_Zr_0.37_O_2_ thin films more accurately than the Cole-Cole and Cole-Davidson equations which have only one distribution parameter. The fitting curves are shown in Figure 16. The fitting parameters of the La_x_Zr_1–x_O_2_ (*x* = 0.09, 0.22, 0.35 and 0.63) dielectrics are provided in Table 1.

From Table 1 and Figure 16, the fitting results of the Cole-Davidson equation showed that the asymmetry of the dielectric loss peak, *β*, increases with decreasing concentration, *x*, of La. To best fit the *x* = 0.09 sample, the width change of the loss peak *α* should be taken into account and, therefore, the HN Equation (15) should be used, where *α* = 0.6535, *β =* 0.3458 and *τ* = 7.3 × 10^−5^ s.

For the fitting of data in the time domain, an empirical expression was proposed by Kohlrausch, Williams and Watts, which is a stretched exponential function, $\text{exp}[-{\left(\text{t}/\tau_{K}\right)}^{\beta_{\text{K}}}]$, [63] to be referred to later as the Kohlrausch-Williams-Watts (KWW) function. The equivalent of the dielectric response function is:

(17) f(t)=dΦ/dt

(18) $$\text{Φ}(t)=\text{exp}[-{\left(\text{t}/\tau_{K}\right)}^{\beta_{\text{K}}}]$$

where the *τ_K_* is the characteristic relaxation time, *β_K_* is a stretching parameter, whose magnitude could vary from 0 to 1. For *β_K_* = 1 the Debye process is obtained. In order to analyze the KWW law in the frequency domain, a Fourier transform is needed. The KWW function in the frequency domain is [63]:

(19) $$\varepsilon *(\omega )=\varepsilon_{\infty }+(\varepsilon_{s}-\varepsilon_{\infty })\int_{0}^{\infty }\beta \tau^{-\beta_{K}}t^{\beta_{K}-1}\text{exp}\left[-{\left(t/\tau \right)}^{\beta_{K}}-i\omega t)\right]dt$$

The KWW law has been widely used to describe the relaxation behavior of glass-forming liquids and other complex systems [64]. The KWW law is not simply an empirical expression, but has a profound theoretical significance. Ngai *et al.* [65,66,67,68] developed a coupling model and derived the Kohlrausch function theoretically. It has already been pointed out by Yoshihara and Work [69] from their careful dielectric measurements that the HN equation can describe the complex permittivity of poly more precisely than the KWW function because the HN equation has two distribution parameters *α* and *β* but the KWW function has only one parameter *β_K_* [70]. However, a possible relationship between *α*, *β* and *β_K_* was hinted at by the results in Reference [71,72,73], where the following analytical relations could be derived:

(20) $$\beta_{K}={\left[(1-\alpha )\beta \right]}^{1/1.23}$$

For characteristic relaxation times, the relationship of *τ* (the relaxation time of the HN equation) and *τ_k_* is

(21) $$\text{ln}(\tau /\tau_{K})=2.6*{\left(1-\beta_{K}\right)}^{0.5}\text{exp}(-3\beta_{K})$$

where *α* and *β* are the distribution parameters of the HN equation and *β_K_* is the distribution parameter of the KWW equation. For the shape parameters, there is a direct transformation from the HN parameters into the KWW parameter. It is well known that a Fourier transform is needed to analyze the KWW law in the frequency domain. However, there is no analytic expression for the Fourier transform of the KWW function in the frequency domain. Any Fourier transform of the KWW function in the frequency domain can be approximated by a HN function which has a more complex relaxation form, but not vice versa [74].

In time domain, the general type of dielectric relaxation can be also described by the Curie-von Schweidler (CS) law (the *t^-n^* behavior, 0 ≤ *n* ≤ 1) [75,76]. After a Fourier transform, the complex susceptibility CS relation is:

(22) $$\chi_{CS}=A{\left(i\omega \right)}^{n-1}$$

where *A* and *n* are the relaxation parameters, *ε_∞_* is the high frequency limit of the permittivity, *χ_CS_ =* [*ε_CS_ ×* (*ω*) − *ε_∞_*]/(*ε_s_* − *ε_∞_*) is the dielectric susceptibility related to the CS law [53]. The value of the exponent (*n*) indicates the degree of dielectric relaxation [63,77]. The values obtained for the exponent *n*, showed that a weak dependence of the permittivity on frequency was observed [78]. A *n−*1 value of zero would indicate that the dielectric permittivity is frequency independent (no dielectric relaxation) [79].

The CS behavior is shown to be faster than the HN function at short times and slower than the HN function at long times. Although the CS relation is empirical, there are many models which link it to physical properties. The majority of these models are based on the presence of compositional or structural inhomogeneities and/or many body effects [80].

To best fit the experimental data, the frequency dependence of complex permittivity ε*(ω) can be combined with the CS law and the KWW law [65]:

(23) $$\varepsilon^{*}(\omega )=\varepsilon_{\infty }+\chi_{CS}(\omega )+\chi_{KWW}(\omega )-i\sigma /(\omega \varepsilon_{s})$$

where *ε_∞_* is the high frequency limit permittivity, *ε_s_* is the permittivity of free space, *σ* is the dc conductivity, *χ_KWW_* = [*ε_KWW_* *(*ω*) − *ε_∞_*]/(*ε_s_* − *ε_∞_*) is the dielectric susceptibility related to the KWW law [53].

**Figure 17:**
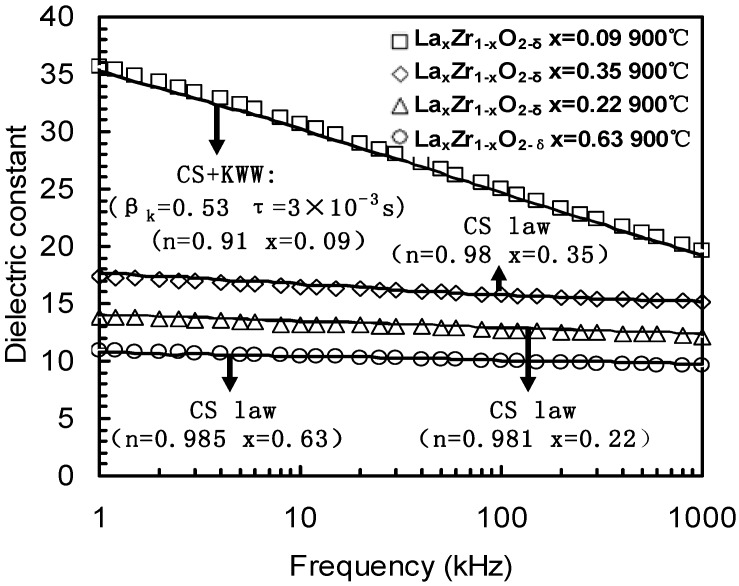
The measured data are the same as Figure 16. All solid lines are fitting results from the CS law or the combined CS+KWW laws. *β_K_*, *τ_K_* and *n* are parameters of the KWW law and CS law [26,32].

The dielectric relaxation data in Figure 16 were modeled by the CS law or the combined CS+KWW laws, as shown in Figure 17. The *k*-values of the La_x_Zr_1−x_O_2_ (*x* = 0.22, 0.35 and 0.63) dielectrics clearly show a power-law dependence on frequency known as the CS law, $k\propto f^{n-1}$, (0 ≤ n ≤ 1) [33,34]. For La_x_Zr_1−x_O_2−δ_ thin films with *x* = 0.63, *x* = 0.35 and *x* = 0.22 La content, the dielectric relaxation response could be ﬁtted by the pure CS law, the *n* values were 0.981, 0.98 and 0.985 when the composition of La, *x*, were 0.22, 0.35 and 0.63, respectively.

However, for the *x* = 0.09 La content, the dielectric relaxation response could not be modeled by the pure CS law or the pure KWW law, but could be modeled by the combined CS+KWW law (Equation (23)). The relaxation parameter *β_k_* and *n* were 0.53 and 0.91, respectively, and the relaxation time *τ_K_* was 3 × 10^−3^ s, as shown in Figure 17. From Figure 17, the exponent value *n* decreased within creasing *k*-values.

Compared with Figure 16, it was found that the combined CS+KWW relaxation process (*β_k_* = 0.53 and *n* = 0.91) could be substituted by the HN function where *α* and *β* were 0.6535 and 0.3458, respectively, in Figure 16 because both the HN function and the combined CS+KWW relationship both have two distribution parameters.

#### 3.2.3. Dielectric Relaxation Mechanisms

**Figure 18:**
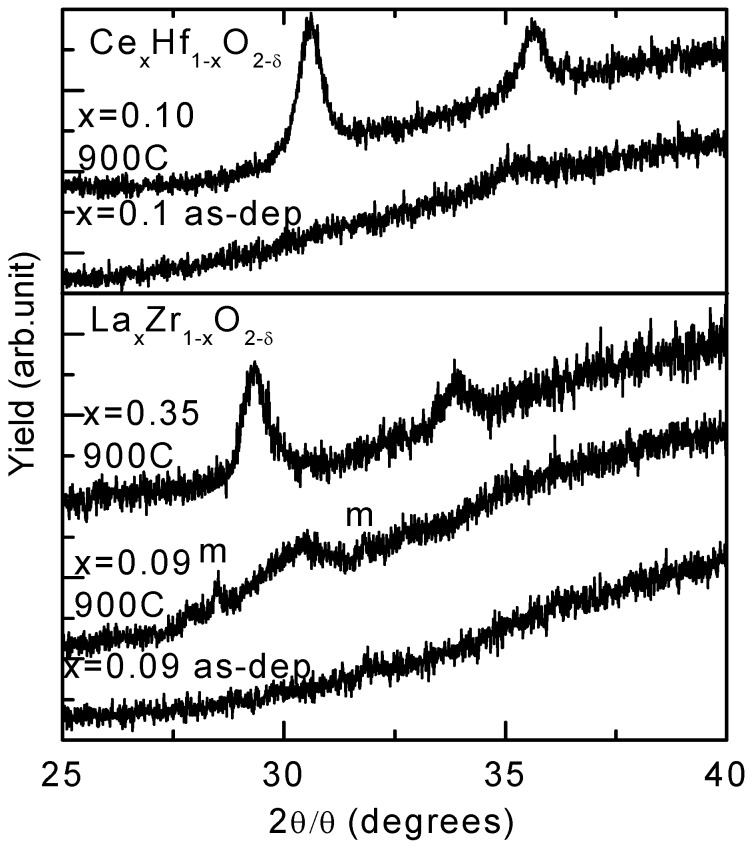
XRD data from La_x_Zr_1−x_O_2−δ_ thin films (bottom) and Ce_x_Hf_1−x_O_2−δ_ thin films (top), as-deposited and following annealing at 900 °C. As-deposited thin films were amorphous. In the annealed thin films, diffraction peaks were from the tetragonal or cubic phase. Data from the monoclinic phase was labeled, *m* [33].

XRD data from several doped thin films are given in Figure 18. All as-deposited samples were amorphous, but became tetragonal or cubic phase after annealing at 900 °C for 15 min. It was clear that a La concentration of *x* = 0.09 stabilized a mixed phase of zirconia of either the tetragonal or cubic phase, with some diffraction features from the monoclinic phase. For the hafnia thin films, Cerium (Ce) doping at a concentration of 10% stabilized the tetragonal or the cubic phase, and no monoclinic features were observed.

Doping hafnia and zirconia thin films with rare earth elements can stabilize the tetragonal or the cubic phase following annealing which enhances the *k*-value [26,34]. The highest *k*-value was obtained with lightly doped thin films, with a doping level of around 10% for both materials, as show in Figure 15 and Figure 16. The level of enhancement was closely related to the doping level. This experimental finding was in close agreement with the predictions of theoretical studies [81,82]. For low levels of doping (~10%): (1) *k*-values of 39 and 33 were obtained from La_0.09_Zr_0.91_O_2−δ_ thin film and Ce_0.1_Hf_0.9_O_2−δ_ thin film, respectively, as shown in Figure 15; (2) the tetragonal or cubic phase was formed, but was accompanied by significant dielectric relaxation. For high levels of doping (such as 35%), (1) no significant enhancement of the *k*-value was achieved; (2) the tetragonal or cubic phase was formed and no significant dielectric relaxation was observed, as shown in Figure 16 [33].

Three possible causes of the dielectric relaxation for the La_x_Zr_1−x_O_2−δ_ dielectric are possible: (1) ion movement of unbounded La^+^ or Zr^+^ ions in the metal-oxide lattice resulting in dielectric relaxation [83]; (2) the combination of unbound metal ions with electron traps, generating dipole moments and inducing dielectric relaxation [84]; (3) a decrease in crystal grain size, causing an increase in the dielectric relaxation due to increased stresses [85,86]. It has been shown that the effect of the cation segregation caused by annealing and rapped electrons on the dielectric relaxation were negligible [26]. However, it has been reported that a decrease in crystal grain size can cause an increase in the dielectric relaxation in ferroelectric relaxor ceramics and this relaxation effect has been attributed to higher stresses in the smaller grains [85,86]. After annealing, the doping level affected the phase of the thin film crystallization and the size of the crystal grains formed that cause the dielectric relaxation. For a La concentration of *x* = 0.35 dielectric thin films with the 900 °C N_2_-annealed containing ~15 nm crystals did not suffer from severe dielectric relaxation and a similar effect appeared to occur with the 900 °C air annealed, producing ~4 nm diameter equiaxed nanocrystallites within the thin film, which suffered from severe dielectric relaxation [87]. So, the cause of the dielectric relaxation is believed to be related to the size of the crystal grains formed during annealing and doping affects the size of the crystal grains formed.

## 4. Conclusions

In summary, in this paper, extrinsic and intrinsic frequency dispersion have been discussed in detail. Two causes of extrinsic frequency dispersion were investigated including the parasitic effect (series resistance, back contact imperfection, cables and connections) and the lossy interfacial layer. These effects were analyzed and modeled based on correction models. Secondly, the surface roughness was observed in ultra-thin dioxide thin films. However, after AFM micrographs were analyzed, the surface roughness was found not to be responsible for the observed frequency dispersion of the thick high-*k* dielectric thin films (>3 nm).

Lastly but not least, after causes for the extrinsic frequency dispersion were considered and determined, intrinsic frequency dispersion (dielectric relaxation) was found to be strongly related to the frequency dependence of the *k*-value on the high-*k* MOS capacitors. For low levels of doping (~10%), *k*-values of 39 and 33 were obtained from the La_0.09_Zr_0.91_O_2−δ_ thin film and Ce_0.1_Hf_0.9_O_2−δ_ thin film, respectively, at 100 Hz; while no significant enhancement of the *k*-value was achieved with high levels of doping (such as 35%).

The dielectric relaxation models in the frequency domain (such as the Cole-Cole equation, the Cole-Davidson equation, the HN equation) and in the time domain (such as the KWW law and the CS law) were comprehensively considered. The dielectric relaxation results of the Ce_x_Zr_1−x_O_2−δ_, LaAlO_3_, ZrO_2_ and La_x_Zr_1−x_O_2−δ_ thin films may be described by either the combined CS+KWW laws or the HN relationship. The fitting results of the HN equation showed that the asymmetry of the dielectric loss peak *β* increases with decreasing concentration levels of La *x*. For a severe dielectric relaxation (for example, the significant decrease of the *k*-value with increasing frequency for the La_0.09_Zr_0.91_O_2−δ_ thin film), the width change of the loss peak *α* played an important role during data fitting. For the La_0.09_Zr_0.91_O_2−δ_ thin film, it was found that the combined CS+KWW relaxation process (*β_k_* = 0.53 and *n* = 0.91) can be substituted by the HN function where distribution parameters *α* and *β* were 0.6535 and 0.3458, respectively because both the HN function and the combined CS+KWW relationship had two distribution parameters.

It was found that dielectric relaxation is related to the size of the crystal grains formed during annealing and that doping affects the size of the crystal grains formed.
